# New Method of Extraction of Cataract

**Published:** 1872-03

**Authors:** 


### New Method for Extraction of Cataract.

The *British Medical Journal* for December 2d contains a clinical lecture by Dr. R. Leibreich, on his new method for cataract extraction. He thus describes it: "The incision of the cornea is to be made with the smallest possible Græfe's knife thus: Puncture and contra-puncture are made in the sclerotic, about one millimetre beyond the cornea, the whole remaining incision passing, with a very slight curve, so that the center of it is about one and a half millimetres distant from the margin of the cornea. This incision can be made upward or downward, with or without iridectomy, and the lens can be removed through it, with or without the capsule." The instruments required are only Græfe's knife, one cystotome, with Daniels' spoon. In three hundred operations the Doctor's per centage of suppuration was not greater than Græfe's method, while the optical results were identical with the best results of flap-extraction.—*Detroit Review of Medicine.*

